# Isolation Transformer Based Very Low Frequency Antenna with Enhanced Radiation Characteristics

**DOI:** 10.1002/advs.202408770

**Published:** 2024-11-28

**Authors:** Jingqi Wu, Zilun Zeng, Liwei Wang, Jianchun Xu, Ke Bi

**Affiliations:** ^1^ State Key Laboratory of Information Photonics and Optical Communications School of Science Beijing University of Posts and Telecommunications Beijing 100876 P. R. China

**Keywords:** impedance matching, low frequency antennas, miniaturization, radiation enhancement

## Abstract

Very low frequency electromagnetic waves adeptly propagate in harsh cross‐medium environments, surmounting the rapid attenuation that impedes high‐frequency counterparts. Traditional low frequency antennas, however, encounter challenges concerning size, efficiency, and power. Here, an isolation transformer based very low frequency loop antenna with compact size and well impedance matching is proposed to enhance radiation characteristics for long‐distance communication. By utilizing the high magnetic permeability amorphous/nanocrystalline alloy Fe_73.5_Cu_1_Nb_3_B_9_Si_13.5_, the isolation transformer enables efficient and stable high‐current input. This design surpasses the current limitation of the conventional loop antennas, thereby improving its electromagnetic performance. Consequently, the radiation intensity of proposed antenna can be effectively increased over 30 times compared to traditional loop antennas. According to the experimental measurements, the transmission distance is over 340 m at 15 kHz using a single proposed antenna with a diameter of 2 m. Its robust performance, such as high radiation efficiency, low power consumption, and long‐distance transmission capacity, suggests significant potential for applications in underground communications and underwater information transmission.

## Graphical abstract

Report a very low frequency antenna with enhanced radiation characteristics, which employs a high‐power‐density, low‐loss amorphous/nanocrystalline alloy Fe_73.5_Cu_1_Nb_3_B_9_Si_13.5_ to fabricate the impedance matching structure, enabling long‐distance low‐frequency communication.

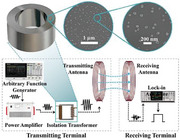

## 1 Introduction

Low frequency (LF, below 300 kHz) electromagnetic waves are known for their low attenuation and strong penetration in transmitting media, which makes them suitable for remote communication, military communication, geological exploration, and so on.^[^ ^1^ ^]^ Underwater communications is one of the main application scenarios of LF electromagnetic waves. In seawater, the attenuation of electromagnetic waves increases rapidly with the increase in frequency. At the same distance, the electromagnetic wave attenuation at 1 MHz reaches more than 160 dB, while the attenuation of the LF electromagnetic wave is only less than 10 dB.^[^ ^2^ ^]^ However, the large size of LF antennas, which often span hundreds or even thousands of meters, presents a significant challenge for the communication system due to the Chu's limit.^[^ ^3^ ^]^ The large physical dimension, intricate feeding network, and substantial power requirement of LF antennas necessitate vast installation space, sometimes covering entire valley. This requirement hinders their deployment in confined spaces, such as mines, tunnels et al., as well as their integration into mobile platforms.^[^ ^4^ ^]^ Hence, the miniaturization of LF antennas holds significant importance for enhancing capabilities in oceanic exploration and advancing underground emergency response and rescue management.

To address the challenges associated with the size and deployment of low‐frequency antennas, various types of potential technologies have been investigated for promoting the miniaturization of low‐frequency antennas. For instance, Xu et al. proposed a mechanical antenna‐based electrostrictive effect of relaxor ferroelectric ceramic (PMN‐PT) to improve radiation capacity and achieve ultra‐wideband characteristics (10 kHz–1 MHz). This design eliminates the dependence on the poled materials and effectively extends the communication distance to 200 m.^[^ ^5^ ^]^ Xiong et al. introduced ferroelectric molecules based on TMCM‐CdCl_3_ and HFPD, exhibiting a significant *d*_33_ coefficient. Due to the similarity in acoustic impedance between molecular ferroelectrics and water, they exhibit lower energy dissipation and longer transmission distances when operating underwater, thereby possessing high application potential in the field of LF underwater communication.^[^ ^6^ ^]^ Hunter et al. demonstrated that extremely LF communications over ≈100 m can be realized in the lower frequency band by a simple, compact, low‐power rotating magnet antenna.^[^ ^7^ ^]^ To further increase the transmission distance at extremely low frequency, Yang et al. developed a magnetomechanical transmission antenna for ELF underwater communication system, achieving an underwater communication distance of 210 m.^[^ ^8^ ^]^ Despite these advancements, the achieved communication distances still remain inadequate for many practical applications.

Apart from the aforementioned antenna designs, the loop antenna stands out as another representative antenna in low‐frequency communications due to its simple structure and excellent transmission performance.^[^ ^9^ ^]^ Except the traditional 100‐meter low‐frequency loop antenna, loop antennas at the meter scale possess short‐range LF communication capabilities. According to Biot–Savart's law, increasing the current in the coil of a loop antenna can effectively amplify the magnetic field component radiated by the antenna, thereby further enhancing its radiation capability. However, the impedance mismatching of the LF loop antennas generally decreases their excitation current capacity, which severely constrains their radiation ability and operating frequency. One effective method to improve the performance of loop antennas at low frequencies is through proper impedance matching. As an electrical component, isolation transformers based on electromagnetic induction have the ability to adjust system impedance.^[^ ^10^ ^]^ This isolation capability is instrumental in resisting noise and interference, making isolation transformers suitable for optimizing impedance matching of loop antennas.

Here, an isolation transformer based VLF antenna is proposed to enhance impedance matching, radiation efficiency, and transmission distance in VLF communications. The isolation transformer acts as the impedance matching structure to boost the input power of the proposed antenna. By adjusting the turn ratio of the isolation transformer, the input impedance can be effectively controlled. For the high energy conversion rate of the isolation transformer, a nanocrystalline material Fe_73.5_Cu_1_Nb_3_B_9_Si_13.5_ with high relative permeability and low coercivity is used to make the magnetic core. The radiation enhancement mechanism of the proposed antenna is analyzed through equivalent circuit models and electromagnetic simulations. Additionally, a VLF wireless communication system, relying on the proposed antenna, is built and achieves over 340 m transmission distance at 15 kHz, verifying the capacity of long‐distance transmission in VLF communications. This isolation transformer based VLF antenna design not only aids in the miniaturization of VLF antennas but also significantly enhances radiation efficiency.

## 2 Antenna Design and Theoretical Analysis

**Figure 1:**
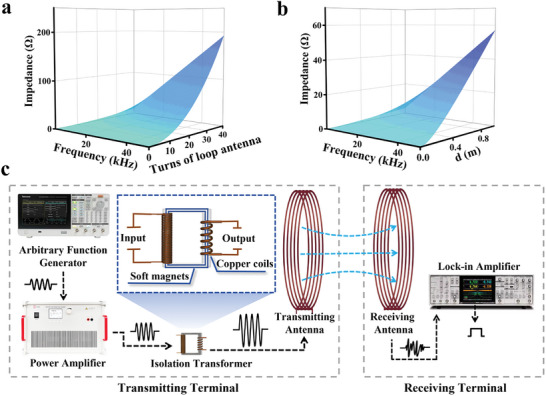
Input impedance of traditional loop antenna with various a) turns and b) diameters. Schematics of c) the proposed VLF communication system.

**Figure** **1**a,b show the simulated impedance of the loop antennas with various radius, turns, and operating frequencies. The simulations indicate a clear monotonic increase in impedance with changes in these parameters. At the desired operating frequency, only the loop antennas with appropriate parameters can realize good impedance matching. In practical applications, most traditional loop antennas are directly connected to a signal source or power amplifier for excitation. Without a specialized processing circuit or wideband design, this direct connection can easily lead to impedance mismatch issues.^[^ ^11^ ^]^ In the VLF band, the power amplifier typically has an output impedance of 50 Ω, whereas the antenna's input impedance is only ≈10^−1^ Ω. When the loop antenna's current reaches the power amplifier's output upper limit, the voltage and output power do not, hindering additional power from entering the antenna. Microwave antennas often adjust impedance by altering their physical structures, such as incorporating tapered baluns. However, these methods result in large physical sizes in low‐frequency applications, preventing antenna miniaturization. Traditional low‐frequency antennas employ resistors, inductors, and capacitors in the construction of impedance matching circuits; nevertheless, the intricacy of these circuits tends to elevate energy transmission losses and augment failure rates. Isolation transformers, commonly used in energy transmission, can also adjust impedance. Compared to traditional methods, high‐power transformers working at VLF are compact, simple, and efficient. By changing the primary and secondary coil turns ratio, the antenna's impedance can be effectively matched.

Therefore, a framework for VLF communication system based on loop antennas with excellent impedance matching has been designed, as illustrated in Figure 1c. The system includes a function generator, power amplifier, isolation transformer, transmitting antenna, receiving antenna, and lock‐in amplifier. Incorporating a transformer increases the loop antenna's impedance to match the power amplifier's 50 Ω. It's equivalent input impedance *Z*_0_ can be represented as:

(1) $$Z_{0}=N^{2}Z_{1}=R_{0}$$

where *N* is the turn ratio of the transformer, *Z*_1_ is the input impedance of the single loop antenna, and *R*_0_ is the output impedance of the power amplifier. According to transformer voltage and current conversion formulas, the transmitting antenna's voltage becomes 1/*N* of the power amplifier's output voltage, while the current increases by *N* times. According to Biot–Savart's law, the generated magnetic field *B*_p_ can be formulated as:^[^ ^12^ ^]^

(2) $$B_{p}=N\frac{r^{2}\mu_{0}nI_{0}}{2{\left(l^{2}+r^{3}\right)}^{3/2}}$$

where *r* is the radius, *n* is the number of turns of the loop antenna, *l* is the transmission distance of magnetic fields, *μ*_0_ is permeability in vacuum, and *I*_0_ is the upper limit current of the power amplifier. The antenna's generated magnetic field intensity is directly proportional to the excitation current *NI*_0_, thus enhancing the radiation intensity by *N* times. And the farthest communication distance can be extended to *N* ^1/3^ times according to the decay equation of magnetic field in free space.^[^ ^4^, ^8^ ^]^ In this system, the radiated signals are received with a loop antenna. However, VLF signal reception is significantly affected by environmental noise, particularly pink noise. To mitigate this interference, the phase‐locked amplifier is employed to pick out weak signals from big environment noise by Fourier transform. After the receiving antenna transforms these signals into an electromotive force, the magnetic field signals are further analyzed in a lock‐in amplifier or spectrum analyzer.

**Figure 2:**
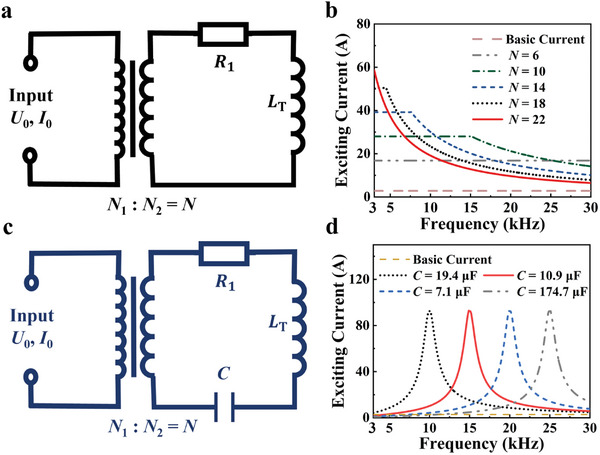
a) Equivalent circuit model of the proposed antenna, and frequency response of b) proposed antenna with different turn ratios. c) Equivalent circuit model of RLC resonant antenna, and d) its frequency response with different capacitance.

An equivalent circuit is constructed to demonstrate the radiation enhancement performance of the proposed isolation transformer based VLF antenna, as shown in **Figure** **2**a. Based on Equation (2), it is evident that the radiation ability of the antenna is proportional to the excitation current *NI*_0_. Thus, the radiation ability of the antenna can be predicted by the level of excitation current. In the simulation setting, the input voltage *U*_0_ is set to 140 V and the input current *I*_0_ is set to 2.8 A, simulating the real parameters of the power amplifier. As shown in Figure 2b, increasing the transformer's turn ratio will multiply the excitation current and lower the resonant frequency. Compared with the basic current of 2.8 A, the highest excitation current at resonant frequency is magnified ≈21 times, reaching 58 A. Moreover, the radiation enhancement also exhibits broadband characteristics, covering the entire VLF band. However, changing turn ratio affects not only the resistance and inductance in the equivalent circuit model but also its capacitance. For more accurate analysis, an equivalent circuit of the RLC resonant antenna is also built as shown in Figure 2c. In the equivalent circuit model of the LF antenna without transformer, the excitation current in the antenna is 2.8 A (named basic current). And the excitation current of the proposed antenna loading the transformer structure, as show in Figure 2d, can reach 90 A at resonant frequencies, which is ≈30 times of the basic current (2.8 A). This implies that the power of transmitting antenna with the same radiation intensity will be greatly reduced by the proposed isolation transformer. Through adjusting the electric parameters, the radiation capacity and operating frequency of the proposed antenna are further optimized, providing more flexibility in our antenna design.

**Figure 3:**
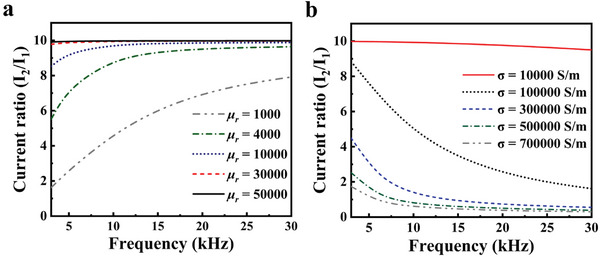
Simulation of a) relative permittivity and b) relative permeability of magnetic core material.

For exploring higher performance, a series of simulations was conducted to investigate the effect of material properties on the transformer's current conversion efficiency, specifically focusing on the magnetic core. As shown in **Figure** **3**a,b, the current conversion efficiency increases with the raised permeability and decreases with the increase of conductivity. According to Bertotti's theory, the power loss *W* of soft magnetic materials is divided into three components: hysteresis loss *W*_h_, classical loss *W*_cl_, and excess loss *W*_exc_. This relationship can be formulated as:^[^ ^13^ ^]^

(3) $$W=W_{h}+W_{\mathrm{cl}}+W_{\mathrm{exc}}$$

(4) $$W_{\mathrm{cl}}=\frac{\pi^{2}}{6}\sigma b^{2}J^{2}f$$

(5) $$W_{\mathrm{exc}}=k_{\mathrm{exc}}\sqrt{\sigma f}J^{3/2}$$

where *k*_exc_ is a parameter related to the properties of the domain structure and their interactions with the structural properties of the material, *J* is peak values of polarization, *b* is thickness, *σ* is conductivity and *f* is the operating frequency of the magnetic core. Based on Equations (4) and (5), using the material with low conductivity for the magnetic core of the isolation transformer can effectively reduce both the excess loss and classical loss of the proposed transformer. And the hysteresis loss *W*_h_ can be substantially decreased by employing higher permeability materials.^[^ ^14^ ^]^ High permeability also typically imparts high power capacity to the transformer.^[^ ^15^ ^]^ The detailed relationship can be represented as:

(6) $$W_{C}=\frac{0.004\mu N_{1}\left(D-d\right)hI_{0}}{\left(D+d\right)}\times \frac{\partial I_{0}}{\partial t}$$

where *W*_c_ is the power capacity of the transformer, *D* is the outer diameter, *d* is the inner diameter, *h* is the height and *μ* is the permeability of the magnetic core, *t* is time, *I*_0_ is the input current and *N*_1_ is the number of turns of the primary coil. Under the condition of a fixed input current, the transformer with a high permeability core exhibits a higher voltage upper limit and larger power capacity. In accordance with the simulated results, the current conversion efficiency of the transformer plateaus when the relative permeability exceeds 30 000 and the conductivity is less than 10 000 S m^−1^.

The properties of various soft magnetic materials, as depicted in **Table** **1**, have been investigated to provide a theoretical basis for selecting magnetic cores in isolation transformers. The relative permeability below 3000 for permendur and ferrites impacts the efficiency of transformer current conversion, while the saturation magnetization below 1 T for permalloy and Co‐amorphous reduces the power density of the magnetic core, hindering its miniaturization. Fe‐based amorphous (AS) alloys based on FeSiB have emerged as a promising class of novel soft magnetic materials developed in recent decades.^[^ ^16^ ^]^ Due to their unique disordered structure, Fe‐based amorphous alloys exhibit lower coercivity *H*_c_ and higher resistivity compared to silicon steel, thus significantly reducing core losses.^[^ ^17^ ^]^ Coupled with their ultra‐low cost, these alloys have become effective alternatives to silicon steel for use as magnetic core materials in transformers operating in the low‐frequency range. In recent years, it has been discovered that high‐temperature‐induced nanocrystallization within amorphous materials can further reduce the material's coercivity *H*_c_ and saturation magnetostriction coefficient while maintaining high permeability, high magnetic flux density, and low conductivity. A low saturation magnetostriction coefficient can minimize mechanical losses due to vibrations during energy transmission through transformers.^[^ ^18^ ^]^ Low *H*_c_ and low conductivity effectively decrease the losses in isolation transformers used in antennas, while high permeability and high saturation magnetization allow for the production of magnetic cores with higher power densities. This facilitates transformers to maintain stable high‐power, high‐current outputs and achieves miniaturization.

**Table 1:** Maximum permeability, coercivity, saturation magnetization, and electrical resistivity of different soft magnetic materials.^[^ ^19^ ^]^

Soft magnetic material	Max. relative permeability [*µm*_ax_]	Coercive field *H*_C_ [A m^−1^]	Saturation polarization *J_S_* [T]	Electrical resistivity [Ωm]
Permalloy	5 × 10^5^	0.4	0.8	70 × 10^−8^
Permendur	2 × 10^3^	100	2.4	40 × 10^−8^
Ferrites	3 × 10^3^	20–80	0.2–0.5	10^−2^–10^5^
Amorphous (Co‐based)	5 × 10^5^	0.5	0.86	120–140 × 10^−8^
Amorphous (Fe‐based)	1.2 × 10^5^	6	1.56	120 × 10^−8^

**Figure 4:**
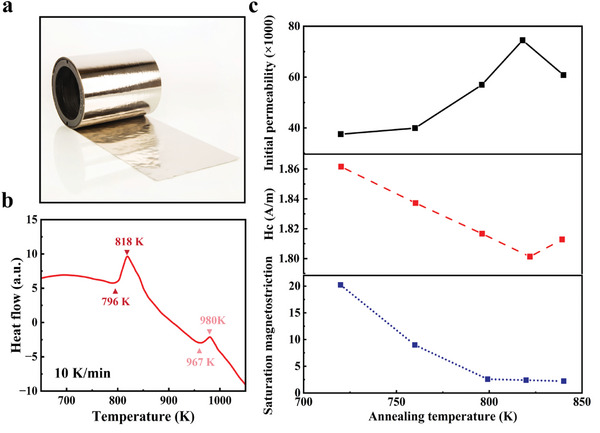
a) Schematic diagram and b) DSC of AS‐Fe_73.5_Cu_1_Nb_3_B_9_Si_13.5_ alloy ribbon. c) The initial permeability, coercivity *H*_c_ and saturation magnetostriction coefficient of Fe_73.5_Cu_1_Nb_3_B_9_Si_13.5_ at different annealing temperatures.

To achieve this goal, the following composition design strategy is adopted: select the appropriate proportion of ferromagnetic elements (Fe) to maximize the local magnetic moment amplitude, maintain the alloy glass forming ability, and add some microalloying elements (Cu, Nb) to form a controllable nanocrystalline structure.^[^ ^20^ ^]^ Based on the research results of Yoshizawa et al., Fe_14.5‐_ *_x_* Cu*_x_* Nb_3_B_9_Si_13.5_ amorphous alloy, when *x *= 1, can obtain higher permeability and lower loss after annealing.^[^ ^16^ ^]^ Based on the comprehensive consideration of cost and performance, a 26 µm thick Fe_73.5_Cu_1_Nb_3_B_9_Si_13.5_ amorphous (AS‐Fe_73.5_Cu_1_Nb_3_B_9_Si_13.5_) alloy ribbon preparation by Advanced Technology and Materials Co,. Ltd was selected as the raw material for making amorphous/nanocrystalline (AN) alloy as shown in **Figure** **4**a. Differential scanning calorimetry (DSC) measurements of the alloy ribbon show a two‐stage crystallization behavior (Figure 4b). In the temperature range of 750–1050 K, the DSC curve shows that the two crystallization exothermic processes are at *T*_x1 _= 796 K and *T*_x2 _= 967 K, respectively. It is worth mentioning that the Fe_73.5_Cu_1_Nb_3_B_9_Si_13.5_ amorphous alloy shows a large Δ*T*_x _= (*T*_x2 _− *T*_x1_) exceeding 161 K, which is much larger than other amorphous alloys and is a benefit for keeping the low cost of manufacturability and good compositional adjustability.^[^ ^21^ ^]^ Based on the DSC result, a heat treatment was conducted to modulate its microstructure. In the introduction to this paper on soft magnetic materials, we report on the testing of initial permeability, coercivity, and saturation magnetostriction coefficients of a material subjected to annealing at various temperatures. These tests were conducted to validate the superiority of AN alloy materials, as illustrated in Figure 4c. Notably, the peak values of both the initial permeability (attaining its maximum) and coercivity (reaching its minimum) were observed at 820 K, which is close to the first crystallization peak at 818 K. Furthermore, the saturation magnetostriction coefficient underwent a rapid decrease from 20 × 10^−6^ to 5 × 10^−6^ after 800 K. Consequently, 820 K was selected as the optimal annealing temperature for AS‐Fe_73.5_Cu_1_Nb_3_B_9_Si_13.5_.

**Figure 5:**
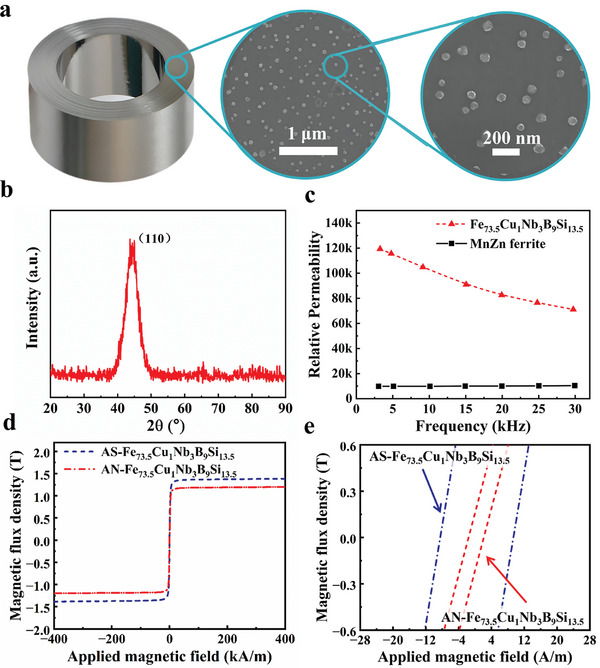
a) SEM and b) XRD of AN‐Fe_73.5_Cu_1_Nb_3_B_9_Si_13.5_ annealing at 820 K. c) Relative permeability of AN‐Fe_73.5_Cu_1_Nb_3_B_9_Si_13.5_ and MnZn ferrite. d) Hysteresis loops and e) its partially enlarged detail of AS‐Fe_73.5_Cu_1_Nb_3_B_9_Si_13.5_ and AN‐Fe_73.5_Cu_1_Nb_3_B_9_Si_13.5_.

**Figure** **5**a displays the results obtained from scanning electron microscopy (SEM), revealing an ultrafine nanocrystalline‐amorphous dual‐phase structure. The nanocrystalline phase comprises uniformly distributed spherical particles ranging from 50 to 100 nanometers in size, embedded within an amorphous matrix. Figure 5b shows the XRD pattern of the prepared AN‐Fe_73.5_Cu_1_Nb_3_B_9_Si_13.5_, featuring distinct crystalline peaks in the 40–50° range. Analysis confirms that the primary component is the bcc‐Fe (Si, B) phase, which arises due to the segregation tendency of Cu and Fe, forming Fe‐rich and Cu, Nb‐rich regions. The Fe‐rich regions serve as nucleation cores for the bcc‐Fe solid solution and undergo selective crystallization. The Cu‐ and Nb‐rich regions surrounding the bcc‐Fe grains are difficult to crystallize due to their higher crystallization temperatures, preventing the growth of bcc‐Fe grains in these areas. Subsequently, other Fe‐rich regions undergo preferential crystallization, resulting in very small grain sizes and a random texture of the alloy. The inductance values of AN‐Fe_73.5_Cu_1_Nb_3_B_9_Si_13.5_ and ferrite are measured using an impedance analyzer. The data is then converted to permeability using the formula:^[^ ^20^ ^]^

(7) $$L=\frac{\mu N^{2}A_{e}}{L_{e}}$$

where *L* is the inductance, *A*_e_ is the effective cross‐sectional area, and *L*_e_ is the effective magnetic circuit length. As illustrated in Figure 5c, Fe_73.5_Cu_1_Nb_3_B_9_Si_13.5_ exhibits a relative permeability exceeding 60 000, significantly higher than that of MnZn‐ferrite. Figure 5d presents the hysteresis loops of the material under different conditions. After annealing at 820 K, the AN‐Fe_73.5_Cu_1_Nb_3_B_9_Si_13.5_ experiences significant improvements in other magnetic parameters while ensuring that the saturation magnetization *B*_s_ does not degrade excessively, remaining above 1.2 T compared with AS‐Fe_73.5_Cu_1_Nb_3_B_9_Si_13.5_. Figure 5e, which is a partial enlargement of Figure 4d, demonstrates the excellent low coercivity *H*_c_ characteristics of AN‐Fe_73.5_Cu_1_Nb_3_B_9_Si_13.5_, reducing the coercivity *H*_c_ from ≈8.5 A m to 2 A m^−1^, effectively lowering the loss during energy transmission when the device serves as an impedance matcher.

The excellent magnetic properties of AN‐Fe_73.5_Cu_1_Nb_3_B_9_Si_13.5_ after annealing (low coercivity, low magnetostriction coefficient, high Bs are presumed to be the result of the combined effects of the following factors: 1) The *B*_s_ of AN alloy is determined by the residual amorphous phase and precipitated phase: *B*_s _= *B*_sc_ *V*_cry _+ *B*_sa_(1 − *V*_cry_), where *B*_sc_ and *B*_sa_ are the magnetic saturation flux densities of the crystalline and amorphous phases, respectively, and *V*_cry_ is the crystalline volume fraction. The internal crystalline volume of AN alloys is extremely small, contributing to a higher *B*_s_ compared to fully crystalline magnetic materials. 2) As the grain size of Fe‐based amorphous alloys decreases, local magnetic anisotropy and magnetostriction performance are reduced, leading to a further decrease in coercivity. Its high saturation magnetic flux density facilitates the use of a compact magnetic core to attain the desired magnetic flux, thereby reducing the transformer's volume and weight. Low coercivity minimizes energy losses during magnetic field changes, boosting transformer efficiency. The minimal magnetostriction coefficient alleviates mechanical stress on the core during such variations, thus enhancing transformer stability and reliability. High permeability improves the core's utilization of the magnetic field, increasing the transformer's power transmission efficiency.^[^ ^22^ ^]^ Consequently, the incorporation of AN‐Fe_73.5_Cu_1_Nb_3_B_9_Si_13.5_ in the fabrication of impedance‐matched isolating transformers holds the promise of achieving compact, efficient, and low‐loss energy transmission within the VLF band.

## 3 Experimental Method and Result

**Figure 6:**
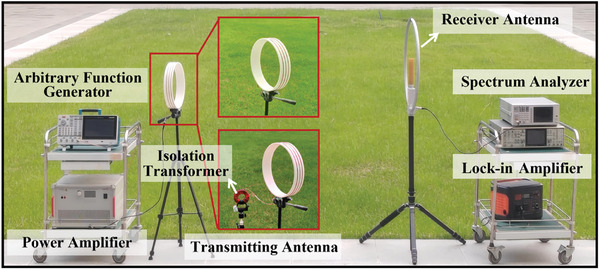
Experimental Set‐up of the VLF Communication System.

The magnetic core, fabricated using the same process as commercial AS alloy cores, was constructed through the stacking of multiple 26 µm AN‐Fe_73.5_Cu_1_Nb_3_B_9_Si_13.5_ thin layers. To mitigate the risk of core saturation during operation, given that the saturation magnetization of AN was only slightly lower than that of AS, the magnet core dimensions were set to a medium size commonly used in the industry of AS alloy (typically work at 400—600 W). Excessively large magnet core dimensions not only increased weight but also posed a risk of heightened eddy current losses. Therefore, an isolation transformer with dimensions of 8 cm outer diameter, 5 cm inner diameter, and 2.5 cm thickness was produced by using 3 cm wide and 26 µm thick AN‐Fe_73.5_Cu_1_Nb_3_B_9_Si_13.5_. As a design example, the desired operating frequency was set to 15 kHz to meet the requirements of VLF band applications. A loop antenna with three turns, a diameter of 30 cm, and a wire length of 5 cm served as the radiation antenna to verify the enhancement characteristics of the proposed structure. Through the analysis of impedance analyzer, the total impedance of the loop antenna at 15 kHz was 0.5 Ω, which was only 0.01 times the output impedance of the power amplifier (50 Ω). According to Formula 1, the turn ratio of the transformer was set to 10:1. The pre‐experiment evaluated the performance of the magnetic cores made of AS‐Fe_73.5_Cu_1_Nb_3_B_9_Si_13.5_ and AN‐Fe_73.5_Cu_1_Nb_3_B_9_Si_13.5_ alloy in the antenna, as shown in Figure S1 (Supporting Information). The transformer made of AN alloy showed higher current conversion efficiency, resulting in a higher excitation current level in the antenna, which improved the radiation efficiency of proposed antenna. Figure S2 (Supporting Information) showed the conversion efficiency of the current using different turns in the case of a turn ratio of 10:1. When the primary coil/coil turns were greater than 100/10, more than 99% of efficient energy transmission could be achieved. Therefore, the number of turns was finally selected as 100:10 to ensure enough power (200 W effective value) was fed into the loop antenna. To evaluate the electromagnetic performance of the proposed antenna, a VLF communication system was built, as shown in **Figure** **6**. At the transmitting terminal, signal processing involved the application of an amplitude shift keying (ASK) modulation scheme. Signal modulation and amplification were carried out by an arbitrary function generator and power amplifier, respectively. By modulating repetition cycles and waveform sequences, a 50% duty cycle ASK signal with a baseband frequency of 5 Hz was generated. The receiving terminal was set at a distance of 6 m from the transmitting terminal. An active loop antenna with a low‐noise amplifier acted as the primary component for signal reception. In the lock‐in amplifier, the signal was filtered by a low‐pass filter to produce an amplified DC output. Appropriately increasing the time constant could effectively improve the signal‐to‐noise ratio of the received signal. Nevertheless, if the time constant was too large, it would lead to signal distortion. Regarding the baseband signal frequency of 5 Hz, a time constant of 3 ms was selected to maintain the integrity of the received signal and ensured a high signal‐to‐noise ratio. The combination of a low‐noise amplifier and a lock‐in amplifier provided a compensatory mechanism, enabling the amplification of minute VLF signals while effectively discerning desired periodic signals. To obtain the accurate frequency response of the proposed antenna, a spectrum analyzer could replace the lock‐in amplifier to analyze the spectral components and power level of received signal.

**Figure 7:**
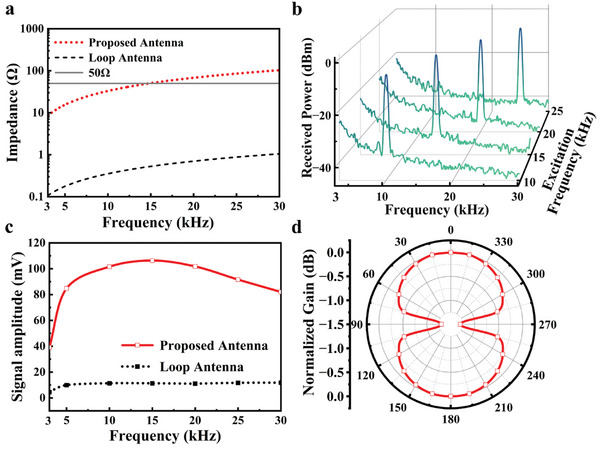
a) Impedance of proposed antenna and conventional loop antenna. b) Received power and c) signal amplitude of proposed antenna with a distance of 6 m. d) Radiation pattern of the proposed antenna.

Through the measurement using an impedance analyzer, the impedances of both two antennas showed an upward trend with increasing frequency, as depicted in **Figure** **7**a. Compared with the conventional loop antenna, the input impedance of the proposed antenna was ≈50 Ω at 15 kHz, achieving good impedance matching. Figure 7b presents the received spectrum when the proposed antenna was stimulated by different feed signals at 10, 15, 20, and 25 kHz. Notably, all received powers at resonant frequencies exceeding −10 dBm, while environment noise remained below −20 dBm. The coincidence of the excitation and reception frequency demonstrated the validity of the VLF communication system in information transmission. The detailed received signals amplitude at different frequencies, as illustrated in Figure 7c, was obtained employing the lock‐in amplifier. Consistent with the principles of impedance matching, the received signals near 15 kHz reached the maximum value and decreased as the frequency deviated from 15 kHz. Moreover, the proposed antenna could achieve ≈10 times signal enhancement at the resonant frequency, which aligned with the results predicted by Equation (2). Figure 7d shows the radiation patterns of the proposed antenna in the axial plane. The radiation patterns exhibited an eight‐shaped profile of distribution, and the maximum radiation occured in the direction of 0° and 180°. At ±45°, the radiation intensity was reduced to half of the maximum value, indicating a 90° primary lobe width of the proposed antenna.

**Figure 8:**
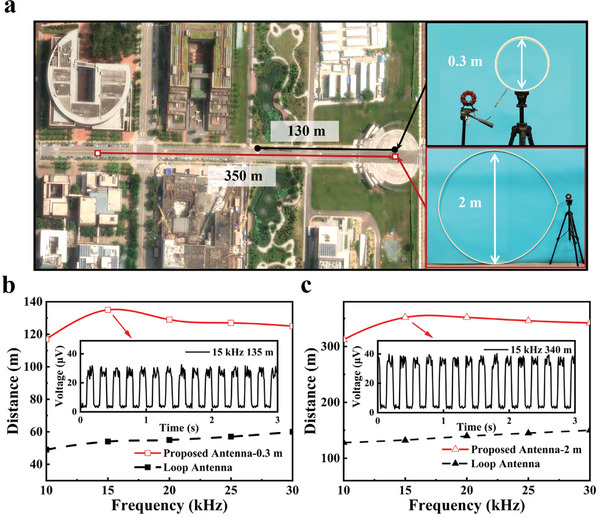
a) Experimental panoramas. b) Transmission distance and modulation signal reception of 0.3 m‐diameter antenna. c) Transmission distance and modulation signal reception of 2m‐diameter antenna.

As shown in **Figure** **8**a, a series of outdoor experiments were carried out on the campus to verify the practical transmission capacity of the proposed antenna. Figure 8b demonstrates that the proposed antenna achieved effective signal transmission up to ≈135 m, whereas the maximum transmission distance of the conventional loop antenna was below 60 m within 10–30 kHz. Additionally, at the resonant frequency of 15 kHz, the logical symbols of the ASK signals were clearly identified at a distance of 135 m. To explore the possibility of increasing the radiation distance, another proposed antenna with a diameter of 2 m was designed. Through detailed calculation based on Equation (1), this larger antenna was wound with a 6.28 m single‐turn wire. Its turn ratio and resonant frequency remain the same as the smaller antenna (i.e., *N *= 10, *f *= 15 kHz). As shown in Figure 8c, the transmission distance of the larger proposed antenna exceeded 300 m, which was more than twice that of a conventional loop antenna with the same size. At the desired frequency of 15 kHz, the effective signal transmission with clear logic levels could reach 340 m. This result indicated that the proposed antenna design offered high flexibility and universality to satisfy the application requirements in different environments.

**Table** **2** compares the proposed antenna with other advanced, miniaturized LF antennas currently reported. Most of the reported antennas were based on the electrostrictive effect of piezoelectric materials or the rotation of permanent magnetic materials, limiting them to narrowband or even single‐frequency communication. This proposed communication method overcame the reliance on piezoelectric and permanent magnetic materials and effectively broadened the working bandwidth while achieving miniaturization. The radiation intensity of 0.45 pT at a distance of 140 m provided by the proposed antenna was at the higher levels among the currently reported low‐frequency antennas. Moreover, its capability to radiate 0.8 pT at 340 m showed its even greater potential. In simulations, a narrow‐band RLC resonant antenna was also tested for greater radiation capability, which could lead to further miniaturization and enhanced radiation.

**Table 2:** Results of signal sending and receiving experiments.

Reference	Core material	Size	Frequency range	Magnetic flux density
[23]	FEP/THV	6 cm	20 Hz	100 pT (0.4 m)
[24]	NdFeB Steel		0–1.6 kHz	50 pT (5 m)
[7]	N42 NdFeB	1.6 cm	100–500 Hz	800 fT (100 m)
[25]	PZT	6 cm	25/35 kHz	100 pT (5 m)
[26]	PZT Metglas	10 cm	30 kHz	1 nT (0.9 m)
[27]	PZT Metglas	10 cm	23.95 kHz	0.1 pT (120 m)
[28]	PZT Metglas Array	6 cm	22.4/24.2/26.3 kHz	1 nT (1.6 m)
[29]	PZT Metglas NiCoil	15 cm	29.46/30.08 kHz	1.2 pT (170 m)
This work	amorphous/nanocrystalline alloys, Cu coil	30 cm	10–30 kHz	0.45 pT (140 m)
		200 cm	10–30 kHz	0.8 pT (340 m)

## 4 Conclusion

An isolation transformer based low frequency antenna with high radiation capacity, frequency tunability, and compact size is proposed. Two equivalent circuit models are employed to clarify the variation in excitation current and its correlation with radiation ability and circuit parameters. In the choice of materials, an amorphous/nanocrystalline alloy Fe_73.5_Cu_1_Nb_3_B_9_Si_13.5_ with a high magnetic flux density, high relative permeability, low coercivity and low saturation magnetostriction coefficient has been constructed as an isolation transformer, enabling more efficient current transmission and ensuring robust support for high‐power input to the antennas. A prototype operating at the resonance frequency of 15 kHz is fabricated, demonstrating a tenfold increase in radiation intensity and more than double the transmission distance. By optimizing the physical parameters of the loop antenna, effective communication over distance exceeding 340 m at 15 kHz is realized. Furthermore, this design methodology is not confined to the VLF band, it shall be general for radiation enhancement across other frequency bands. Optimizing the antenna Geometry can yield higher radiation intensity and extend transmission distance. This flexible and low‐cost design method expands the application potential of loop antennas in VLF communication and advances the development of miniaturized VLF antennas.

## Conflict of Interest

The authors declare no conflict of interest.

## Supporting information



Supporting Information

## Data Availability

The data that support the findings of this study are available from the corresponding author upon reasonable request.
