# Supplementary material for: Isolation Transformer Based Very Low Frequency Antenna with Enhanced Radiation Characteristics
Source: Adv Sci (Weinh). 2024 Nov 28;12(3):2408770. doi: 10.1002/advs.202408770 (PMC11744638; doi:10.1002/advs.202408770)
Supplement: Supplementary file 1 — Supporting Information [file ADVS-12-2408770-s001.docx]

Supporting Information

Isolation Transformer based Low Frequency Antenna with Enhanced Radiation Characteristics

*Jingqi Wu, Zilun Zeng, Liwei Wang, Jianchun Xu* and Ke Bi**


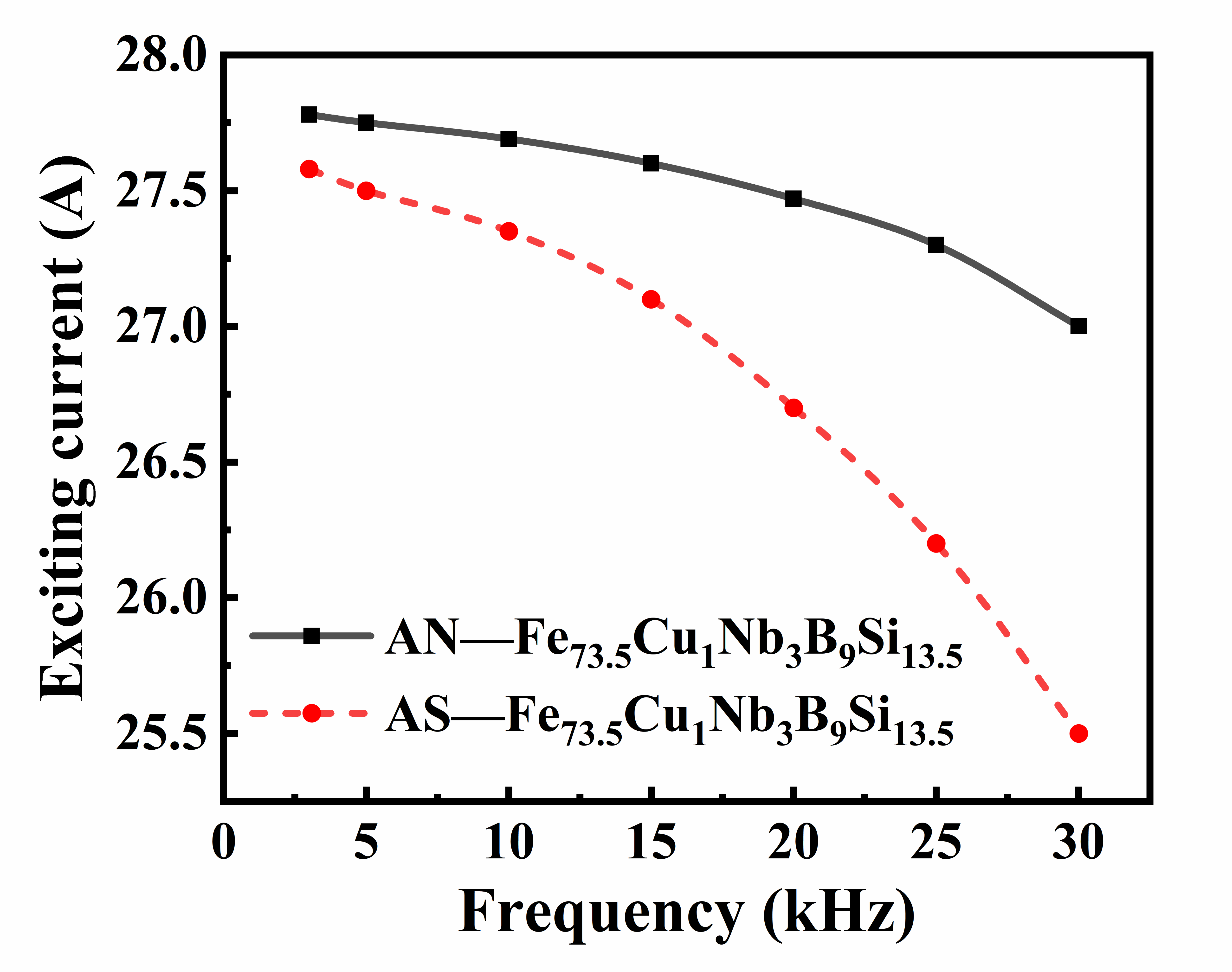


Figure S1. The exciting current in the loop antenna when AS-Fe_73.5_Cu_1_Nb_3_B_9_Si_13.5_ and AN-Fe_73.5_Cu_1_Nb_3_B_9_Si_13.5_ alloy are used as the magnetic cores of the isolation transformer.


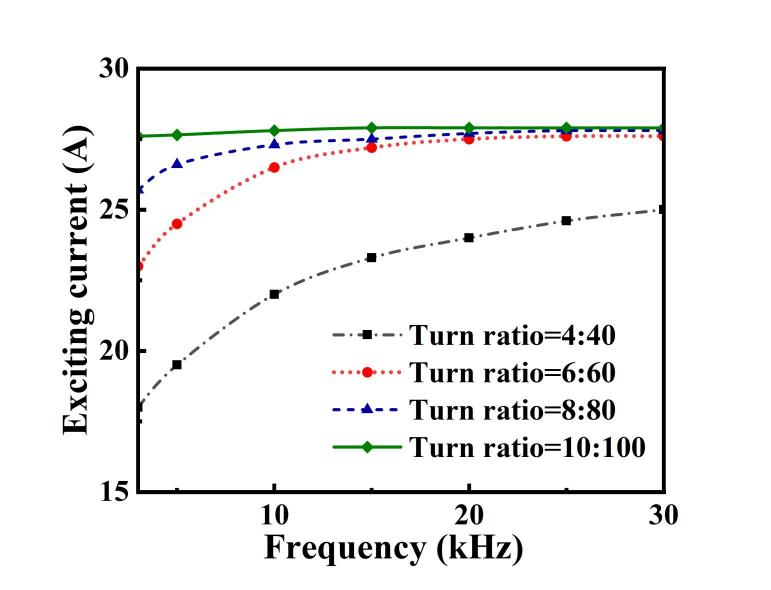


Figure S2. Exciting current in loop antenna with the transformer in different turns.
